# PlantDB – a versatile database for managing plant research

**DOI:** 10.1186/1746-4811-4-1

**Published:** 2008-01-08

**Authors:** Vivien Exner, Matthias Hirsch-Hoffmann, Wilhelm Gruissem, Lars Hennig

**Affiliations:** 1 Institute of Plant Sciences & Zurich-Basel Plant Science Center, ETH Zurich, CH-8092 Zurich, Switzerland

## Abstract

**Background:**

Research in plant science laboratories often involves usage of many different species, cultivars, ecotypes, mutants, alleles or transgenic lines. This creates a great challenge to keep track of the identity of experimental plants and stored samples or seeds.

**Results:**

Here, we describe PlantDB – a Microsoft^® ^Office Access database – with a user-friendly front-end for managing information relevant for experimental plants. PlantDB can hold information about plants of different species, cultivars or genetic composition. Introduction of a concise identifier system allows easy generation of pedigree trees. In addition, all information about any experimental plant – from growth conditions and dates over extracted samples such as RNA to files containing images of the plants – can be linked unequivocally.

**Conclusion:**

We have been using PlantDB for several years in our laboratory and found that it greatly facilitates access to relevant information.

## Background

Research in many plant biology laboratories involves cultivation of plants. Often, these plants can have different genetic composition. Work uses different species, cultivars, ecotypes, mutants, alleles, transgenic lines and others. Because most higher plants produce viable, but dormant seeds, which can be stored for prolonged times, plant research often involves a much larger diversity of lines than animal research. Several recent reverse genetic initiatives, mainly in Arabidopsis and rice [1,2], powerful transformation protocols [3,4] and easy genotyping assays have led to a massive increase in lines used by the average plant science laboratory. Although this creates great opportunities for research, it also creates a great challenge to keep track of the identity of experimental plants. This challenge is faced by geneticists, physiologists, biochemists and molecular biologists alike.

Although databases are commonly used by biologists to store diverse data such as vector information [5] or microarray data [6], databases are much less used to make work with plants more efficient. Here we present a Microsoft^® ^Office Access database called PlantDB that was designed to store data of experimental plants. The goal was to develop a database solution that is easy to use, adjustable to specific needs and available to many colleagues.

## Implementation

**Figure 1:**
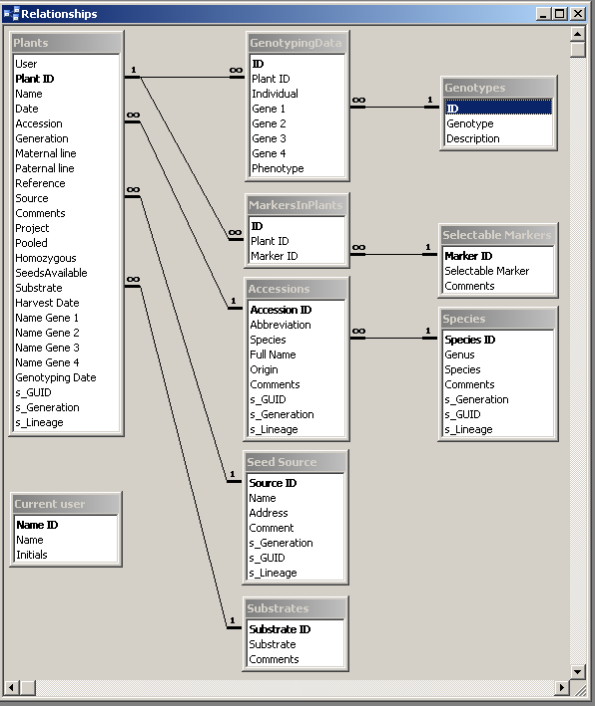
**Architecture of PlantDB**. Shown are the tables and their relations in PlantDB.

**Figure 2:**
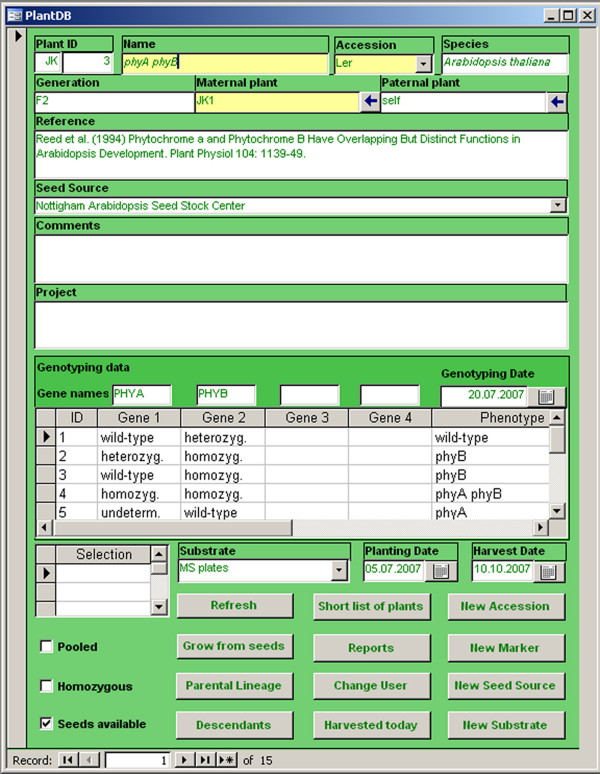
Screenshot of the main user interface of PlantDB.

Because the Microsoft^® ^Office software suite is commonly used by many plant scientists, we developed PlantDB using Microsoft^® ^Office Access. At the heart of the database there are 10 tables for data storage and selection. Figure 1 shows these tables and the relations between them. The main way the user communicates with the database is through predefined forms, and the *PlantDB *form is the main entry point (Fig. 2). Together there are 10 forms for data entry and retrieval. For documentation purposes, we included 8 predefined reports that can be used to create hard copies of parts or the entire database content. Finally, there is Visual Basic for Applications (VBA) code for specific tasks such as search for parental lines.

## Results and discussion

### Uses and application of PlantDB

PlantDB was designed to be used in the following way. Whenever a researcher prepares plants for cultivation, plants derived from the same parents and subjected to identical treatments on the same day will receive a unique identifier (provided automatically by PlantDB). Sibling individuals will get individual sub-identifiers separated by a dash from the main identifier. For example, plants JK1-1 and JK1-2 are sibling plants under identical treatments while plants JK1-1 and JK2-1 are plants (i) from different parents, (ii) under different treatments (such as soil versus plates), or (iii) from identical parents under identical treatments but sown on different days. The idea is that the unique identifiers will be used to label plates, pots and derived samples, such as extracted RNA, and to refer to the plants in laboratory notebooks. In addition, if any seeds are harvested they will be stored under the identifier of the mother plant.

### Single-user versus multi-user scenarios

By default, PlantDB uses user initials as part of the unique plant identifiers. To personalize the empty PlantDB framework, users need to change the name and initials of the current user by clicking the *Change user *button. This way, each laboratory member can set up his/her own database and store the relevant information. Alternatively, laboratories could decide to use one common database for all members. In this case, name and initials of the current user could be set to any name such as the group leader's or the gardener's. It is currently not possible for different collaborators to use their personal initials but store the data in one common database. However, future developments of PlantDB are planned to include this option.

### Data entry into PlantDB

If a new database entry is created three types of information must be provided (fields highlighted in yellow):

*1. Name*. This field holds the name of the plants (e.g. mutant allele or line identifier). Entries can be freely chosen.

*2. Accession*. This field holds accession, cultivar or sub-species information. Its value must be selected from a predefined list. Selection of the appropriate accession or cultivar will automatically fill the species field as well. If the used accession or cultivar is not in the list, the *New Accession *button can be used to add new accessions/cultivars and connect them to the corresponding species. If the species is not yet available, it can be added to the database using the *New Species *button. Note, that PlantDB is not restricted to be used for a single species such as *Arabidopsis thaliana*, but can hold data for plants of any number of species.

*3. Maternal plant*. This field holds information about the mother plant. Because this field is mandatory, a value such as "unknown" has to be entered if no information is available. The identifier of the mother plant can be freely chosen, but usage of the naming convention defined here will enable vertical searches through the database later (see below).

In addition to the required three fields, these optional fields are available for data entry:

*4. Generation*. This field can hold information on the generation, such as F1 or T2.

*5. Paternal plant*. This field can hold information about the father plant. As for the *Maternal plant *field, usage of the naming convention defined here will enable vertical searches through the database later (see below).

*6. Reference*. This field can hold identifiers from seed stock databases such as NASC [7] and details of relevant publications.

*7. Seed source*. This field holds information on the origin of the used seeds. The default is the name of the current user, but other values can be selected from a list. The *New Seed Source *button can be used to add other seed sources to the database.

*8. Project*. This field can hold details about experiments these plants will be used for such as RNA-extraction or bulking up seeds.

*9. Comments*. This field can hold any free text. However, the main purpose is to store observations made during plant cultivation such as unusual phenotypes or pathogen infections.

*10. Genotyping*. This section can hold information on genotyping for up to four loci. First, the *Gene Name *field can hold the names of the tested genes, and the *Genotyping date *field can hold the date of genotyping for the purpose of cross-reference with laboratory notebooks. Dates can easily be entered using a calendar form. Second, for an arbitrary number of sibling plants the genotypes for each of the up to four tested loci can be selected from a list of predefined values. In addition, comments on phenotypes can be stored.

*11. Selection*. This field holds information about selectable markers. Entries need to be selected from a list of predefined values. The *New Marker *button can be used to add new markers to the database.

*12. Substrate*. This field holds information about the growth substrate such as soil, sand or medium for in vitro cultivation. Entries need to be selected from a list of predefined values. The *New Substrate *button can be used to add new cultivation substrates to the database.

*13. Planting date*. This field holds information on sowing or planting dates and is filled automatically with the current date when a new entry is created.

*14. Harvest date*. This field can be filled when seeds are harvested.

*15. Pooled*. This tick box specifies whether seeds from multiple sibling plants were pooled at harvest.

*16. Homozygous*. This tick box specifies whether plants are homozygous for a mutation or transgene.

*17. Seeds available*. This tick box specifies whether seeds from these plants are available. It can be set active at harvest and inactive once seeds are used up.

### Simplified data entry

When starting to cultivate new plants, filling the database with all required and optional values can become time-intensive. If seeds are used that come from a plant documented in the same database, data entry can be made easier: after the identifier of the mother plant and/or the father plant has been entered, the *Grow from seeds *button will start a search for entries of parental plants in the database. If present, name, accession, reference, homozygosity state and selection markers will be copied from the parental entries. If a generation was specified for the mother plant, it will be incremented (e.g. F1 will be changed to F2). If paternal plant is not self, generation is set to F1. Values for name, reference and selection marker are combined from mother and farther plants.

To accelerate entry of harvesting dates, the *Harvested today *button can be used. Clicking this button will enter the current date into the harvest date field.

### Data analysis – pedigree searches

When working with several generations or when backcrossing mutants, it is often desirable to establish pedigree trees. Two buttons (*Parental lineage *and *Descendants*) allow to rapidly search from the current record upwards (i.e. for parents, grandparents etc.) or downwards (i.e. for children, grandchildren etc.).

In addition, common functionality of MS Office Access can be used to quickly query the database such as for entries with a particular mutant in homozygous state for which seeds are available.

### Generating hard copies of data base contents

For documentation and backup purposes, reports can be created. Most importantly, the *Short list of plants *report generates a table with some of the most often used information. A hard copy of this table can kept in the laboratory for quick reference.

## Conclusion

PlantDB provides an easy way to efficiently store information about experimental plants. Some laboratories might have particular requirements for types of data about experimental plants to be stored, and the open architecture in MS Office Access makes it very easy to adapt PlantDB to such specific requirements. Identifiers defined in PlantDB can be used to label files containing images of plants or derived samples. This allows unequivocally linking of all information about any experimental plant – from growth conditions and dates over extracted derived samples to files containing images of the plants. We have been using PlantDB for several years in our laboratory and found that it greatly facilitates shared access to relevant information.

## Availability and requirements

• **Project name: **PlantDB

• **Project home page: **

• **Operating system(s): **Microsoft Windows 2000 or higher

• **Other requirements: **Microsoft ^® ^Office Access 2000 or higher, Visual Basic for Applications

• **Any restrictions to use by non-academics: **none

PlantDB is freely available from the journal's and the authors' web pages (see also Additional file 1). It can be used, modified and distributed freely as long as this publication and the original authors are acknowledged. If research projects benefited much from PlantDB, this publication should be cited in arising papers.

## Competing interests

The author(s) declare that they have no competing interests.

## Authors' contributions

LH designed and programmed the software and drafted the manuscript. VE participated in design and programming the software and drafted the manuscript. MHH participated in programming the software. WG helped to draft the manuscript. All authors read and approved the final manuscript.

## Supplementary Material

Additional file 1 **Installation package for PlantDB**. This file contains an empty version of PlantDB, which can be used immediately, and an example database, which can be examined for reference purposes. Unpack into any directory. Opening requires MS Office Access installed on the PC. Note that the pedigree search function of PlantDB requires the MSHFlexgrid ActiveX library. If using the pedigree search function results in an error message because this library is not installed on the user's computer, run the included "ActiveX_for_PlantDB01.MSI" file. This file requires administrator rights for successful installation. Click here for file
